# Overexpression of miR-669m inhibits erythroblast differentiation

**DOI:** 10.1038/s41598-020-70442-y

**Published:** 2020-08-11

**Authors:** Ryutaro Kotaki, Masaharu Kawashima, Asuka Yamaguchi, Naoto Suzuki, Ryo Koyama-Nasu, Daisuke Ogiya, Kazuki Okuyama, Yuichiro Yamamoto, Masako Takamatsu, Natsumi Kurosaki, Kiyoshi Ando, Akihiko Murata, Masato Ohtsuka, So Nakagawa, Koko Katagiri, Ai Kotani

**Affiliations:** 1 Department of Hematological Malignancy, Institute of Medical Science, Tokai University, Isehara, Kanagawa Japan; 2 Division of Clinical Oncology and Hematology, The Jikei University School of Medicine, Minato-ku, Tokyo, Japan; 3 Department of Biosciences, School of Science, Kitasato University, Sagamihara, Kanagawa Japan; 4 Department of Computational Biology and Medical Sciences, Graduate School of Frontier Sciences, The University of Tokyo, Kashiwa, Chiba Japan; 5 Department of Hematology and Oncology, Tokai University School of Medicine, Isehara, Kanagawa Japan; 6 Division of Immunology, Department of Molecular and Cellular Biology, School of Life Science, Faculty of Medicine, Tottori University, Yonago, Tottori Japan; 7 Department of Molecular Life Science, Division of Basic Medical Science and Molecular Medicine, Tokai University School of Medicine, Isehara, Kanagawa Japan; 8 Biomedical Informatics Laboratory, Department of Molecular Life Science, Tokai University School of Medicine, Isehara, Kanagawa Japan; 9 Micro/Nano Technology Center, Tokai University, Hiratsuka, Kanagawa Japan; 10 Precursory Research for Embryonic Science and Technology, Japan Science and Technology Agency, Saitama, Japan; 11 AMED-PRIME, Japan Agency for Medical Research and Development, Tokyo, Japan

**Keywords:** Erythropoiesis, miRNAs

## Abstract

MicroRNAs (miRNAs), one of small non-coding RNAs, regulate many cell functions through their post-transcriptionally downregulation of target genes. Accumulated studies have revealed that miRNAs are involved in hematopoiesis. In the present study, we investigated effects of miR-669m overexpression on hematopoiesis in mouse in vivo, and found that erythroid differentiation was inhibited by the overexpression. Our bioinformatic analyses showed that candidate targets of miR-669m which are involved in the erythropoiesis inhibition are A-kinase anchoring protein 7 (Akap7) and X-linked Kx blood group (Xk) genes. These two genes were predicted as targets of miR-669m by two different in silico methods and were upregulated in late erythroblasts in a public RNA-seq data, which was confirmed with qPCR. Further, miR-669m suppressed luciferase reporters for 3′ untranslated regions of Akap7 and Xk genes, which supports these genes are direct targets of miR-669m. Physiologically, miR-669m was not expressed in the erythroblast. In conclusion, using miR-669m, we found Akap7 and Xk, which may be involved in erythroid differentiation, implying that manipulating these genes could be a therapeutic way for diseases associated with erythropoiesis dysfunction.

## Introduction

Blood cells, such as lymphocytes, myelocytes, and erythrocytes, are produced and are replenished continuously for a lifetime from hematopoietic stem/progenitor cells through a multi-step differentiation process called hematopoiesis. The blood cells have vital functions, e.g. immunological protection and O_2_/CO_2_ transfer. While the hematopoiesis is indispensable, a dysregulation in the process causes hematological malignancies. Hence, a proper modification of the hematopoiesis is possibly a rational treatment for hematological malignancies and other diseases induced by a dysregulation in the hematopoiesis.

Previous studies revealed that microRNAs (miRNAs) are one of crucial factors for regulating hematopoiesis^1^. miRNAs are 18–25 nt single-stranded non-coding RNAs which post-transcriptionally downregulate gene expression through RNA-induced silencing complex^2, 3^. Each miRNA possesses several target mRNAs, and a computational prediction implied that targets of total miRNAs account for more than a third of all human genes^4^. Indeed, miRNAs are involved in many biological processes and phenomena, such as cell differentiation, embryogenesis, and tumorigenesis^2^. In terms of the hematopoiesis, it has been reported that overexpression of miR-150 resulted in blocking B cell development at the Pro-B to Pre-B cell transition through downregulation of Myb, among other targets^5^. miR-181a downregulated various phosphatases modulating sensitivity of T cells to antigen peptides, and overexpression of miR-181a in hematopoietic stem/progenitor cells increased fraction of B-lineage cells^6, 7^. In line with these studies, we have found several miRNAs which can modulate hematopoiesis and can improve hematologic malignancy^8–11^.

Here, we studied miR-669m in the context of the hematopoiesis. We had firstly picked the miR-669m as one of candidate miRNAs for another study, in which subsequent experiments omitted this miRNA from the list of the candidates, remaining functions and target genes of the miR-669m unidentified (K.O. and R.K. unpublished data). The miR-669m gene is included in a miR-466-669 gene cluster. This cluster is the third largest miRNA gene cluster in mice and is located on intron 10 of the Sfmbt2 gene, which encodes a polycomb group protein and has been reported critical for trophoblast maintenance^12^. Cell differentiation process, such as hematopoiesis, is accompanied with dynamic changes in a chromatin structure landscape. Hence, it is possible that the miR-466-669 gene cluster is expressed in such processes simultaneously with its parental Sfmbt2 gene and affects the hematopoiesis. Hence, we investigated effects of miR-669m on the hematopoiesis.

In the present study, we transferred miR-669m-transduced hematopoietic stem/progenitor cell fraction into recipient mice and analyzed hematopoietic cell populations differentiated from the transduced cells. We found that the miR-669m-transduced cells were impaired in erythroid differentiation. Using in silico prediction of miR-669m target genes in combination with a published gene expression profile of erythroid cells, we further found two miR-669m target genes, Akap7 and Xk, which are possibly responsible for the miR-669m-induced erythropoiesis impairment. The present study may have implications that we can regulate erythropoiesis through manipulating miR-669m and/or the two target genes.

## Results

### miR-669m overexpression inhibits on erythropoiesis in vivo

**Figure 1:**
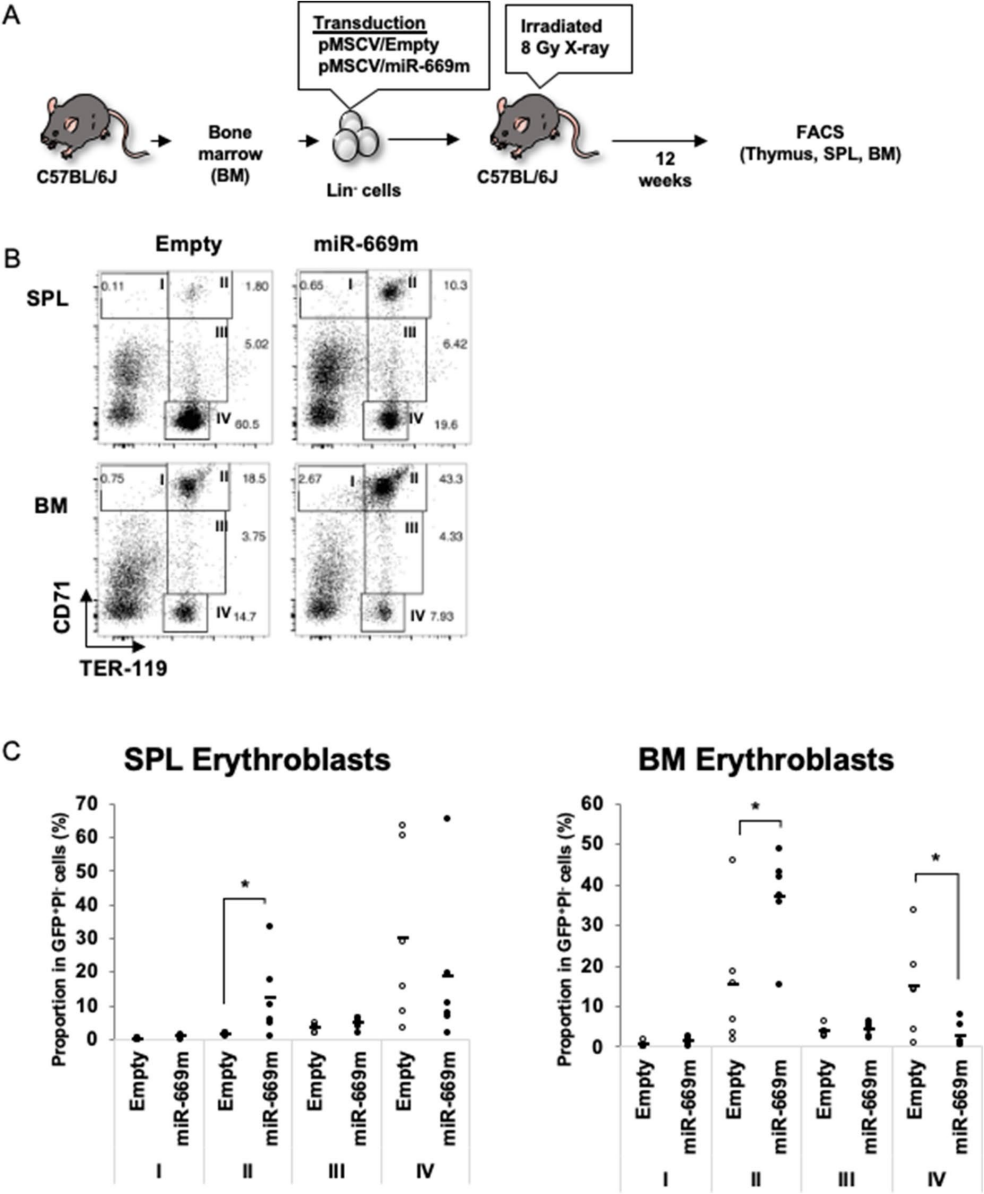
miR-669m overexpression inhibits erythroid differentiation in vivo. (**A**) A procedure of bone marrow (BM) transplantation is shown. BM Lin^−^ cells were transduced with pMSCV/Empty or pMSCV/miR-669m vectors. One day after the transduction, the cells were intravenously injected into recipient C57BL/6 J mice which had been irradiated with 8 Gy X-ray one day before. After 12 weeks, spleen (SPL) and BM cells were analyzed by FACS. (**B**, **C**) Representative plots (**B**) and pooled data (**C**) of CD71/TER119 expression on SPL and BM GFP^+^PI^−^ cells were described. In (**C**), each dot represents data from one recipient mouse (n = 6). The horizontal bars describe mean. Statistical analysis was performed using Student’s *t* test (*p < 0.05).

To investigate effects of miR-669m on hematopoiesis, hematopoietic stem /progenitor cells isolated from mouse bone marrow (BM) were transduced with retroviral expression vector of miR-669m with GFP label (pMSCV/miR-669m), which were subsequently transferred into sublethal X-ray irradiated recipient mice (Fig. 1A). miR-669m overexpression from this vector was confirmed by transfection of HEK293T cells (Fig. S1). After 12 weeks from the transfer, we analyzed hematopoietic stem cell population among GFP^+^ miR-669m-transduced cells in BM, spleen, and thymus with flow cytometry. Proportions of T cell progenitors in the thymus and mature CD4 and CD8 T cells in the spleen showed no significant difference between miR-669m-transduced cells and control vector-transduced cells (Fig. S2). Similarly, Pro-B (B220^+^IgM^−^CD43^+^CD19^-^), PrePro-B (B220^+^IgM^−^CD43^+^CD19^+^), Pre-B (B220^+^IgM^−^CD43^−^CD19^+^), and immature B (B220^+^IgM^+^) cells in the BM and mature/transitional B cells (B220^+^IgM^+^) in the spleen had no difference (Fig. S3). These results suggest that neither T nor B cell differentiation were affected by miR-669m overexpression. Among myeloid cell populations, CD11b^high^Gr1^+^F4/80^-^ granulocyte proportion in miR-669m-transduced cells in the BM were increased compared with the control, although no difference in myeloid cells was observed in the spleen (Fig. S4). Further, we found differences in erythroblast populations between miR-669m- and control vector-transduced cells. The erythroblasts are classified into four subsets, CD71^high^Ter119^med^ (I), CD71^high^Ter119^high^ (II), CD71^med^Ter119^high^ (III), and CD71^low^Ter119^high^ (IV), as previously described (Fig. 1B)^13^. Erythroblast differentiation proceeds in the order of the numbers (from the region I to the region IV). The region I mainly consists of proerythroblasts. The region II includes basophilic erythroblasts. The region III includes late basophilic and chromatophilic erythroblasts. The region IV is mainly orthochromatophilic erythroblasts. In the BM, miR-669m-transduced cells showed higher proportion of the region II cells and lower proportion of the region IV cells than the control vector-transduced cells (Fig. 1B, C). The result suggests that miR-669m overexpression inhibits erythroblast differentiation from the region II to the region III, resulting in accumulation of the region II cells. The region III cells showed no difference between the samples probably due to very low frequency of this population, which might indicate that this population quickly differentiates into the region IV cells. In addition, erythroblasts with the same phenotype as the region II cells were observed in the miR-669m-transduced cells in the spleen. Since this population in the spleen was rarely observed in the control, the region II phenotype cells in the miR-669m-transduced cells in the spleen might be region II cells which leaked from the BM. Collectively, miR-669m overexpression inhibits the late erythroblast differentiation.

### In silico prediction identified three candidate target genes of miR-669m suppressing erythropoiesis

**Figure 2:**
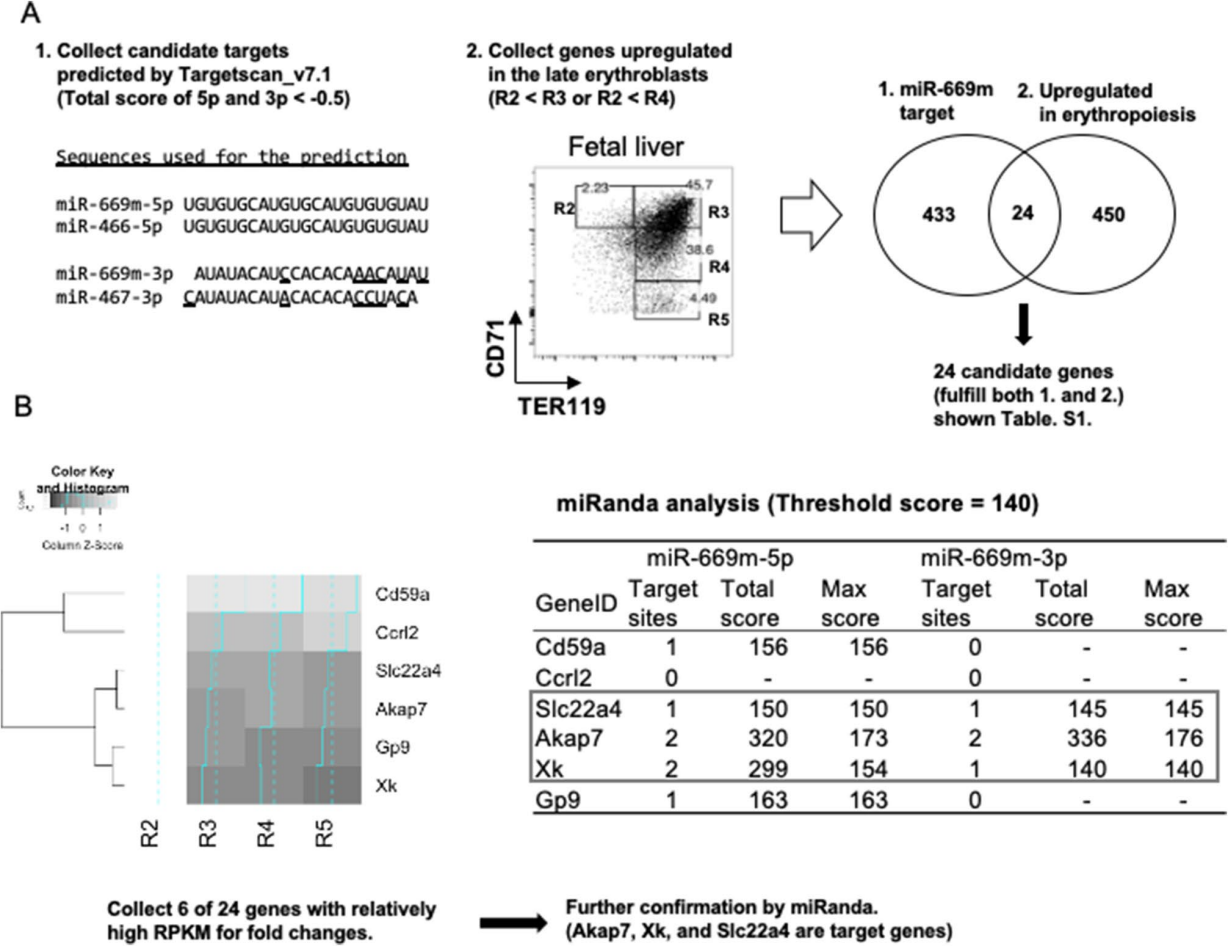
Three candidate target genes of miR-669m involved in erythroid differentiation were identified by bioinformatics analyses. (**A**) Targetscan predicted targets of miR-669m-5p and miR-669m-3p using miR-466-5p and miR-467-3p sequences, respectively. We should note that the sequences of miR-669m-3p and miR-467-3p are not identical. We collected 457 candidate genes targeted by miR-669m with total score of 5p and 3p < −  0.5 (1). On the other hand, we reanalyzed published RNA-seq data of E14.5 FL erythroblast populations. CD71/TER119 plot from our experiment is presented to show the R2 to R5 populations analyzed in the published data. Genes upregulated in R3 and/or R4 compared with R2 were picked up (474 genes) (2). We identified 24 candidate genes overlapping between (1) and (2) (listed in Table S1). (B) We focused on six genes with relatively high RPKM (reads per kilobase of exon per million mapped sequence reads) among the candidate 24 genes (left: Heatmaps). Further, miRanda analysis was performed with threshold score 140, and we selected three genes, Akap7, Xk, and Slc22a4 as candidate genes (right).

We next explored the target genes of miR-669m which are responsible for its inhibitory effect on erythropoiesis. First, we listed predicted target genes of miR-669m using Targetscan program^14^. Two mature miRNAs, miR-669m-5p and miR-669m-3p, produced from miR-669m, were analyzed. In this analysis, we used the sequences of miR-466-5p and miR-467-3p instead of the miR-669m products, which was automatically performed by the program because of their same or similar sequences. We should note that the miR-467-3p sequence is not the same as the miR-669m-3p, whereas the miR-466-5p and the miR-669m-5p have the identical sequence. As a result, we obtained 457 candidate target genes which had a total prediction score of the 5p and 3p less than − 0.5 (Fig. 2A: left). Genes reported to control late erythroid differentiation, such as *Gata1, Gata2, Scl/Tal1, Fog, Lmo2, Ldb1, Klf1, Riok3, Mxi1, Gcn5, Myb*, and *Hdac2*^15, 16^ were not included in the targets of miR-669m (Scores shown > − 0.05 or not applicable), indicating that these genes are not involved in the miR-669m-mediated erythropoiesis inhibition (Table S2).

To obtain genes involved in the erythroblast differentiation, we analyzed previously published RNA-seq of fetal liver (FL) erythroblast cells (Fig. 2A: right). In the data, erythroblasts were classified into the same four populations as the present study using CD71 and TER119 expression^17^. We hypothesized that miR-669m downregulates some genes which need to be upregulated in the late erythroblasts for further differentiation. We obtained a list of 474 genes which are upregulated in R3 and/or R4 compared with R2.

Subsequently, we analyzed overlap between the predicted miR-669m target genes and the genes upregulated in the late erythroblasts and identified 24 overlapping genes (Fig. 2A, Table S1, and Dataset S1). Among the 24 genes, six genes which showed relatively high reads per kilobase of exon per million mapped sequence reads (RPKM) and fold changes in the published data were selected for the next step (Fig. 2B: left). We further analyzed miR-669m targeting scores for the six genes in another miRNA target prediction program, miRanda, using the exact sequences of miR-669m-5p and -3p with 3′ UTR sequences of the six genes obtained from the NCBI gene database. From these analyses, we finally obtained three candidate genes whose 3′ UTRs contain multiple target sites and relatively high targeting scores for miR-669m as follows: A-kinase anchoring protein (Akap7), X-linked Kx blood group (Xk), and solute carrier family 22 member 4 (Slc22a4) (Fig. 2B: right).

**Figure 3:**
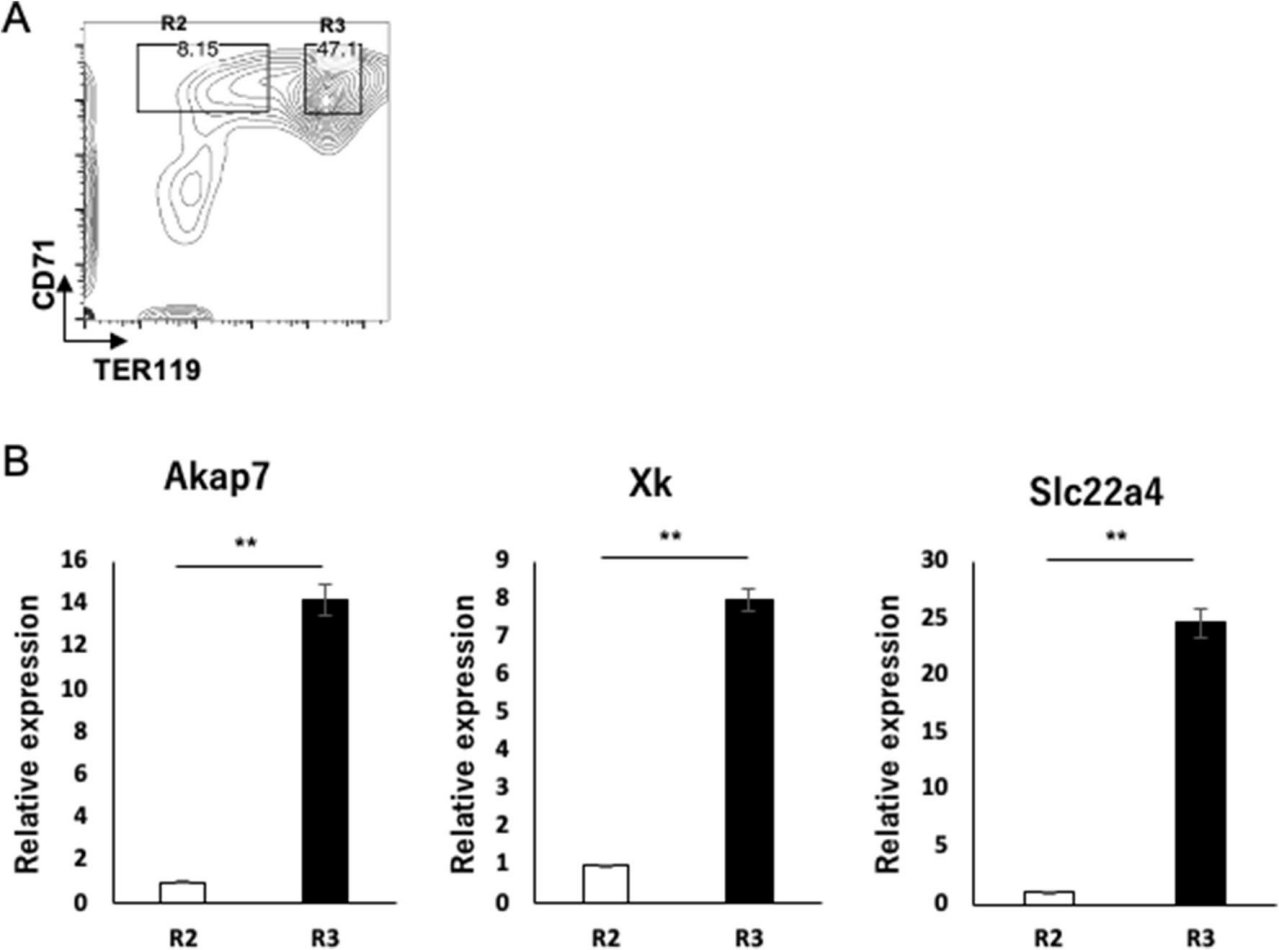
qPCR analyses confirmed that the candidate genes are upregulated through progression of erythroid differentiation. (**A**) E13.5 or E14.5 FL cells were collected and erythroblasts were enriched by MACS. The erythroblast-enriched cells were stained with CD71/TER119, and the R2 and R3 fractions were sorted for subsequent qPCR. (**B**) qPCR was performed for candidate target genes of miR-669m. The expression level was normalized to that of Gapdh gene as an internal control. The graphs describe mean ± SD (*n* = 3). Statistical analysis was performed using Student’s *t* test (**p < 0.01). Representative data of two independent experiments are shown.

We next confirmed that the target genes are indeed upregulated in the late erythroblasts. qPCR analyses of FL cells revealed that these candidate genes are upregulated in CD71^high^TER119^high^ cells compared with CD71^high^TER119^low^ cells (Fig. 3). We also analyzed two other published RNA-seq data of FL cells^18, 19^, and confirmed that those genes were similarly elevated as erythroid differentiation progressed (Fig. S5).

### Akap7 and Xk are targets of miR-669m

**Figure 4:**
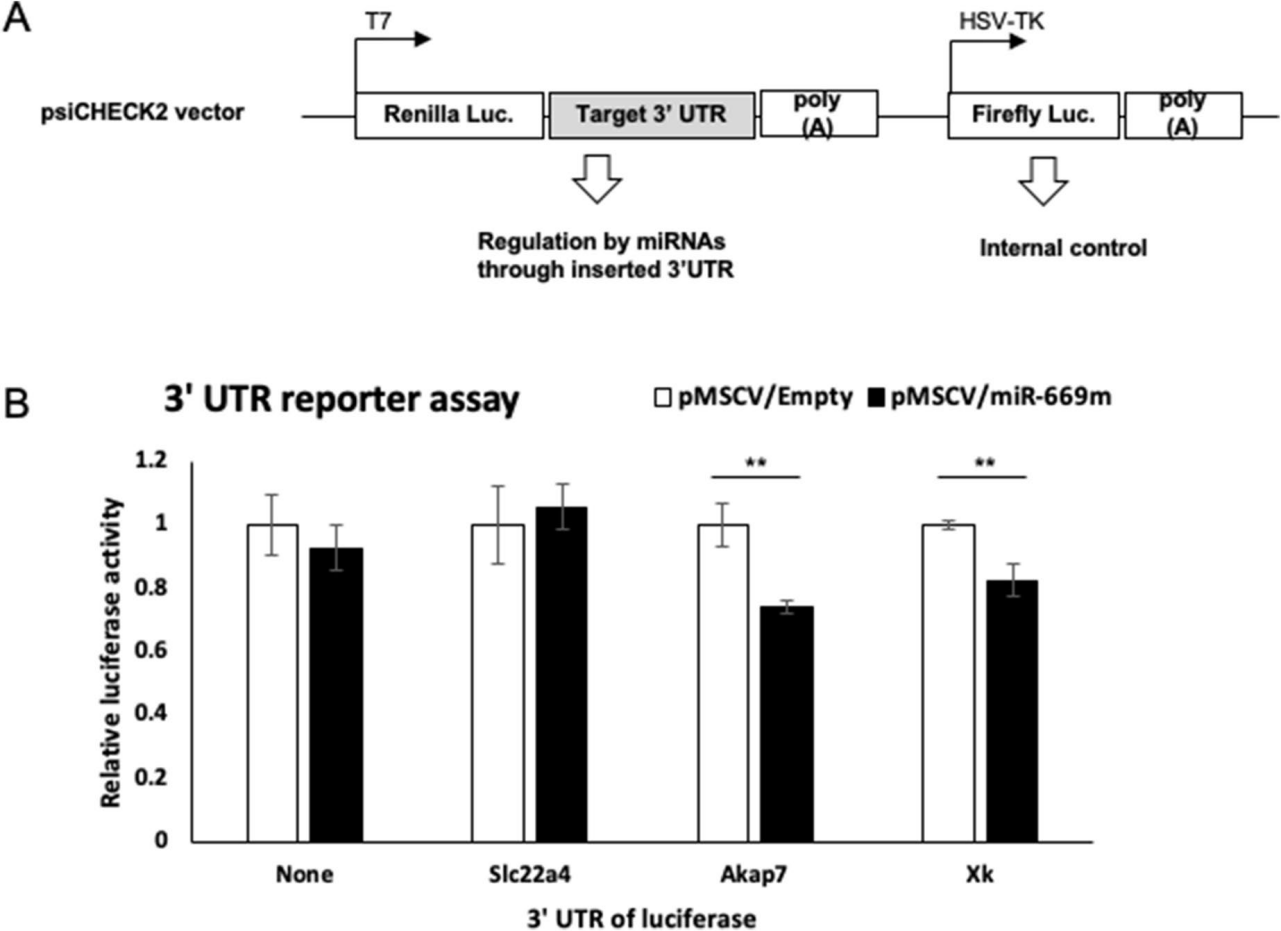
Akap7 and Xk are targets of miR-669m. HEK293T cells were cotransfected with luciferase reporter for 3′-UTR of target genes and miRNA expression vectors, and then dual luciferase assay was performed. (**A**) Construction of the dual luciferase is shown. 3′-UTRs of target genes (Akap, Xk, and Slc22a4) were cloned into downstream of Renilla luciferase gene, which results in that the luciferase mRNA can be regulated by miRNAs through the 3′-UTRs. The plasmid also includes independently regulated firefly luciferase gene, which can be used as an internal control. (**B**) Relative luciferase activity of pMSCV/Empty and pMSCV/miR-669m were presented. Vertical axis is relative luciferase activity (fold change to pMSCV/Empty). The Renilla/firefly luciferase ratio was assessed and normalized to the control. The graphs describe mean ± SD (n = 3). Statistical analysis was performed using Student’s *t* test (**p < 0.01). Representative data of two independent experiments are shown.

We further tested suppressive effects of miR-669m on 3′-UTRs of the candidate genes, Akap7, Xk, and Slc22a4, using a luciferase reporter system. For this assay, we prepared plasmid vectors in which 3′- UTRs of the genes were inserted into the downstream of the Renilla luciferase gene (Fig. 4A). These vectors were transfected into HEK293T cells simultaneously with pMSCV/miR-669m or control pMSCV/Empty vector, and the Renilla luciferase activity was measured. The values were normalized to activity of firefly luciferase, which is also encoded in the reporter vector as an internal control transcribed from an independent constitutive promoter. Compared with the pMSCV/Empty vector, pMSCV/miR-669m suppressed the activity of the luciferase encoded with 3′-UTR of Akap7 and Xk genes (Fig. 4B). These results suggest that miR-669m directly downregulates Akap7 and Xk through 3′-UTRs of these genes. On the other hand, miR-669m had no effect on the 3′-UTR of Slc22a4 gene.

**Figure 5:**
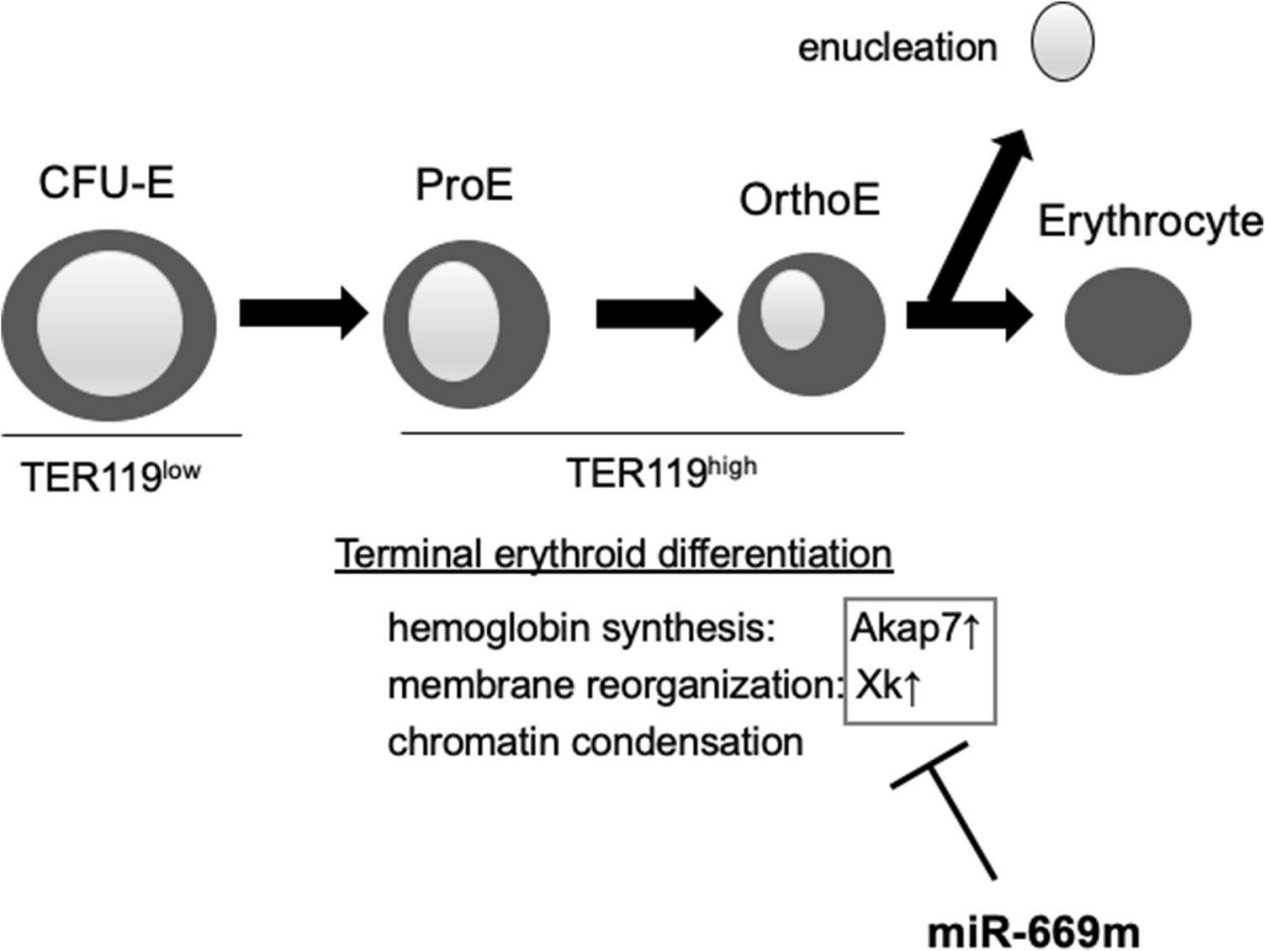
Schema for miR-669m suppressing erythroid differentiation. Colony-forming unit-erythroid (CFU-E) differentiated to proerythroblasts (ProE), and then orthochromatophilic erythroblasts (OrthoE) which undergo enucleation. miR-669m inhibits Akap7 and Xk, which are probably involved in terminal erythroid differentiation.

Taken together, we have concluded that Akap7 and Xk genes probably suppressed by miR-669m overexpression. The miR-669m overexpression, which suppresses Akap7 and Xk genes, inhibited late erythroblast differentiation, while late erythroblasts in the physiological state upregulated Akap7 and Xk genes, suggesting that these genes are involved in the late erythroblast differentiation. Akap7 gene encodes a scaffold protein of protein kinase A (PKA)^20^. PKA is essential for heme biosynthesis in the process of erythropoiesis^21^. Xk encodes a component of red blood cell membrane complex, which might relate to membrane reorganization of erythrocyte^22^. These previous studies support the idea that Akap7 and Xk genes play important roles in erythroblast differentiation (Fig. 5).

We also examined physiological expression of miR-669m in the process of erythroblast differentiation. The three public datasets of RNA-seq, we analyzed for gene expression in Fig. 2 and Fig. S5, were analyzed for miRNA expression. All of the datasets revealed virtually no or very low expression of miR-466-669 cluster miRNAs in the erythroblasts (Tables S3–S5). Although miR-466f-3p showed relatively high expression among the cluster miRNAs, one of the miR-466f gene, miR-466f-4, is not located in the cluster and hence is regulated separately, which might have contributed to the relatively high expression. Similarly, we should also note that miR-466i, which showed the highest expression among miRNAs in all of the datasets, is not a member of the miR-466-669 cluster. In addition to the miRNAs of the miR-466-669 cluster, expression of the parental gene, *Sfmbt2*, was also quite low in the erythroblasts (Tables S6–S8). These results suggest that miR-466-669 cluster is not expressed in the physiological erythroblasts, and only few fractions might exist in the cells at those stages. This is consistent with the idea that miR-669m is not expressed in the physiological erythroblasts, otherwise it may disturb the differentiation process of the erythroblasts.

## Discussion

Here, we found that overexpression of miR-669m inhibited erythroid differentiation in mice. Our in silico prediction suggested that this inhibition was mediated by suppression of Akap7, Slc22a4, and Xk genes. Further, luciferase reporter assay for 3′-UTRs of the candidate target genes revealed that miR-669m indeed has suppressive effects on 3′-UTRs of Akap7 and Xk genes. These genes were upregulated in the late erythroblast fractions. Collectively, our results suggest that Akap7 and Xk may be involved in erythroid hematopoiesis.

Erythropoiesis mainly consists of early erythropoiesis, terminal erythroid differentiation, and reticulocyte maturation^23^. In the present study, miR-669m overexpression inhibited the terminal erythroid differentiation. The terminal erythroid differentiation refers to differentiation process from proerythroblasts (CD71^high^TER119^med^), through basophilic (CD71^high^TER119^high^) and, polychromatophilic (CD71^med^TER119^high^) erythroblasts, to orthochromatophilic (CD71^low^TER119^high^) erythroblasts, which subsequently undergo enucleation. miR-669m overexpression probably inhibited differentiation from CD71^high^TER119^high^ basophilic erythroblasts to CD71^med^TER119^high^ polychromatophilic erythroblasts (Fig. 1). During the terminal erythroid differentiation, many changes occurs, e.g. decreased size of erythroblasts, hemoglobin synthesis, membrane reorganization, chromatin condensation, and enucleation occured^16, 23^. Hence, the miR-669m target genes, Akap7 and Xk, may be involved in these processes.

Akap7 encodes a scaffold protein for PKA. A previous study showed that an inhibitor for adenylyl cyclase, which is an upstream enzyme of PKA, inhibits erythroid differentiation of human leukemia cell line^24^. Further, another PKA scaffold, AKAP10, is important for heme biosynthesis in human and mouse^21^. Akap7 might be also important for erythroid differentiation as a PKA scaffold. The other suggested target, Xk encodes a protein which has been reported to be a component of a multiprotein complex involved in erythrocyte membrane and cytoskeleton interaction^22^. Xk deficiency leads to McLeod syndrome, which has been reported to be accompanied with morphological and functional damages of red blood cells^25^. These reports suggest that Xk might play an important role in membrane reorganization, which is one of the processes involved in terminal erythroid differentiation. Collectively, the miR-669m target genes we found, which are upregulated in the late erythroblasts, might be involved in the terminal erythroblast differentiation (Fig. 5). Further studies are needed for involvement and detailed functions of Akap7 and Xk genes in the terminal erythroblast differentiation.

miR-669m is a member of miR-466-669 cluster, which is involved in cell proliferation and apoptosis and promotes mouse placenta formation^26, 27^. In addition, miR-669a and miR-669q have been reported to prevent skeletal muscle differentiation through MyoD suppression^28^. In the present study, miR-669m overexpression inhibited erythroblast differentiation. In the physiological erythroblasts, most of the miR-466-669 cluster members and the parental *Sfmbt2* gene, which are uniformly regulated at transcription, were barely or not detected. This suggests that miR-669m gene, included in the cluster, is not expressed in the physiological erythroblast stages, consistently with the inhibitory effects of miR-669m on the terminal erythroblast differentiation. Since the expression of miR-669m was already low at the early stage (TER119^−^) of the differentiation and was unchanged throughout the differentiation process, miR-669m suppression may not be sufficient for the terminal erythroblast differentiation in the physiological state. In addition to the erythroblasts, we also found that miR-669m overexpression increased CD11b^high^Gr1^+^F4/80^−^ granulocyte in the BM. More detailed analysis would be needed to reveal mechanisms underlying the enhancement of the granulocyte fraction.

The analyses on the RNA-seq data of the physiological erythroblasts showed that miR-669m-5p was still detectable at low level, whereas miR-669m-3p was completely not. We confirmed miR-669m-5p, but not -3p, was barely detectable in qPCR (data not shown). In contrast, the overexpression vector probably induces expression of both miR-669m-5p and -3p (Fig. S1). In addition, our miRanda prediction showed that both arms have target sites in the 3′ UTRs of genes. Hence, we could not determine which arm is responsible for the suppression of the identified target genes.

In the present study, we identified the genes which may be involved in the erythropoiesis, using miRNA overexpression and target prediction to narrow down the candidates. Our results suggest that suppression of Akap7 and Xk genes by miR-669m overexpression probably inhibits erythropoiesis. Although we should note that mouse and human have some differences in transcriptomic profiles through terminal erythroid differentiation^29, 30^, Akap7 and Xk may play an important role not only in mouse but also in human erythropoiesis as discussed above. The present study suggests that miR-669m and these target genes are potential therapeutic targets. For example, inhibition of the target genes by miR-669m or specific inhibitors could be physically less burdened therapy for polycythemia vera than phlebotomy, especially in elder patients. Further studies are needed to assess miR-669m and its targets for therapeutic applications.

## Methods

### Bone marrow transplantation

BM cells were obtained from 8- to 10-weeks-old female C57BL/6 J mice (CLEA, Tokyo, Japan). Subsequently, Lin^−^ cells were enriched using MACS isolation as described below. The Lin^−^ cells were transduced with a miR-669m overexpressing or empty retroviral vectors and were intravenously transplanted into recipient C57BL/6 J mice, which had been irradiated with 8 Gy X-ray one day before the transplantation. The recipients were dissected 12 weeks after transplantation, and thymus, SPL, and BM cells were analyzed.

### MACS isolation

To enrich Lin^-^ cells from BM or FL cells, the whole cells after red blood cell lysis were stained with biotin anti-mouse Gr1 (RB6-8C5, BioLegend), biotin anti-mouse/human B220 (RA3-6B2, BioLegend), biotin anti-mouse TER-119 (TER-119, BioLegend), biotin anti-mouse/human CD11b (M1/70, BioLegend), and biotin anti-mouse CD3ε (145-2C11, BioLegend). Then the cells were labeled with anti-biotin magnetic MicroBeads (Miltenyi Biotec). The unlabeled cells were enriched using AutoMACS (with “depletes” program; Miltenyi Biotec). To enrich erythroblasts, the same procedure was performed except that biotinylated TER119 was excluded from the label.

### Construction of plasmids

Precursor of miR-669m-1 was cloned into the multiple site of pMSCV-proIRES2-GFP (Addgene) vector using Xhol and Notl restriction enzymes. 3′ UTRs of Akap7, Xk, and Slc22a4 genes were cloned into multiple cloning site of psiCHECK2 (Promega) vector using XhoI and NotI restriction enzymes. The plasmids were prepared and amplified using DH5α competent cells (Invitrogen).

### Retroviral transduction

To prepare retroviral vectors, pMSCV plasmids were transfected into Plat-E retroviral packaging cells using X-tremeGene 9 DNA (Sigma) according to the manufacture’s instruction. Two days after, supernatant was harvested and was filtrated through 0.45 μm filters, and subsequently polybrene was added (8 μg/mL at final concentration).

The vectors were transduced into BM Lin^-^ cells which were cultured with TPO, SCF, and Flt3L for 24 h before the transduction or freshly isolated FL Lin^-^ cells. The cells were suspended in the viral vector suspension in flat bottom 24 well plates. The plates were centrifuged for 99 min at 32 °C, 2,500 rpm, and were subsequently incubated for 2–3 h at 37 °C. After that, the cells were harvested, were washed with medium for three times, and were cultured at 37 °C. Transduction efficacy was checked as GFP^+^PI^−^ cell proportion using FACS analysis.

### Flowcytometry

The following antibodies were used: APC anti-mouse CD71 (R17217, BioLegend), PE anti-mouse TER119 (TER119, BioLegend), PE/Cy7 anti-mouse TER119 (TER119, BioLegend), APC anti-mouse CD4 (GK1.5, BioLegend), PE/Cy7 anti-mouse CD8α (53-6.7, TONBO Biosciences), PE anti-mouse CD3e (145-2C11, BioLegend), APC anti-mouse IgM (II /41, eBioscience), PE/Cy7 anti-mouse B220 (RA3-6B2, BioLegend), APC/Cy7 anti-mouse CD19 (6D5, BioLegend), PE anti-mouse CD43 (eBioR2/60, eBioscience), APC/Cy7 anti-mouse CD11c (N418, BioLegend), PE anti-mouse CD11b (M1/70, BioLegend), PE/Cy7 anti-mouse Gr-1(Ly-6G/Ly-6C) (RB6-8C5, BioLegend), APC anti-mouse F4/80 (BM8, eBioscience), FITC streptavidin (BioLegend).

Cells were analyzed using FACS Verse (BD Biosciences). Dead cells were excluded by PI (propidium iodide, Sigma) staining. Data were analyzed by FlowJo software (Tree Star). FL CD71^high^TER119^low^ or CD71^high^TER119^high^ cells were sorted by FACS Melody (BD Biosciences).

### qPCR

Total RNA was isolated using TRIzol (Invitrogen). cDNAs were synthesized using.

High Capacity cDNA Reverse Transcription Kit (Applied Biosystems) and by miScript II RT kit (Qiagen) for mRNA and miRNA, respectively. Real-time qPCR was performed using the Fast SYBR Green Master mix with StepOnePlus real-time PCR system (Applied Biosystems). Threshold cycle (CT) values were calibrated to those of internal controls, Gapdh (for mRNAs) or RNU6 (for miRNAs), and analyzed by the 2^−ΔΔCT^ method.

Sequences of primers are as follows:

- Akap7.
- Forward: GCAGATGGAGACCATGTTAGC.
- Reverse: ATGCCTTTTTCCCGAAATGTTC.
- Xk.
- Forward: CAGTTCACGCTCCTCTTCG.
- Reverse: GATACAAAAGACTTCACAACACCTG.
- Slc22a4.
- Forward: GGAACATTGCCACCATAACC.
- Reverse: AGAGCAAAGTAACCCACTGAGG.
- Gapdh.
- Forward: AACTTTGGCATTGTGGAAGG.
- Reverse: ACACATTGGGGGTAGGAACA.
- qPCR of miRNA.
- miR-669m-5p Forward: TGTGTGCATGTGCATGTGTGTAT.
- miR-669m-3p Forward: ATATACATCCACACAAACATAT.
- RNU6 Forward: CGCAAGGATGACACGCAAATTC.
- Universal Reverse Primer: GAATCGAGCACCAGTTACGCA.

### In silico prediction of miR-669m target genes

For in silico prediction of targets of miR-669m, we used TargetScanMouse_v7.1 website^14^ and miRanda algorithm as described previously^31^.

### Reanalysis of open RNA-seq data

We obtained the published RNA-seq data that are deposited into the Gene Expression Omnibus (GEO) database with accession number GSM796041-44 (E14.5 FL cells sorted by CD71/TER119)^17^, GSM640429-31 (E14.5 or E15.5 FL cells sorted as burst-forming unit (BFU-E), CFU-E, and TER119^+^)^18^, and GSM1859519-20 (E14.5 FL cells sorted as TER119^-^ and TER119^+^)^19^. For RNA-seq samples of these data, raw sequences were mapped to the mouse reference genome (GRCm38) using HISAT2^32^. Next, the gene expressions were evaluated by transcripts per kilobase million (TPM) using StringTie^33^.

To examine the expression of miR-669m in normal conditions in red blood cells, we examined the raw sequences from RNA-seq data of GSM1859519-20, GSM640429-31, and GSM1859519-20. We applied Magic-BLAST to map the short reads to the mature miRNA sequences obtained from the miRbase^34^. We then counted the fraction of mapped reads to each miRNA using SAMtools with an idxstats option^35^.

### Dual luciferase assay

HEK293T cells were co-transfected with 20 ng psiCHECK-2 vector (Promega) and 100 ng pMSCV/miR-669m or pMSCV/Empty vector in 96 well plate. After cultured for 24 h, Renilla and firefly luciferase activity were measured as previously described^9^. The Renilla/firefly luciferase ratio was calculated and normalized to the control.

### Statistical analysis

Student’s *t* test was performed to determine the significance of the differences. *p* value < 0.05 were defined statistically significant. All analyses were performed by Microsoft Excel.

### Study approval

All animal procedures and protocols were reviewed and approved by the Animal Care Committee of Tokai University, and all experiments were performed in accordance with the relevant guidelines and regulation.

## Supplementary information

Supplementary Information 1.

Dataset S1
