# Supplementary material for: Overexpression of miR-669m inhibits erythroblast differentiation
Source: Sci Rep. 2020 Aug 11;10:13554. doi: 10.1038/s41598-020-70442-y (PMC7419302; doi:10.1038/s41598-020-70442-y)
Supplement: Supplementary file 1 — Supplementary Information 1. [file 41598_2020_70442_MOESM1_ESM.pdf]

Supplementary Information for

Overexpression of miR-669m inhibits erythroblast differentiation

Ryutaro Kotaki[1)], Masaharu Kawashima[1, 2)], Asuka Yamaguchi[1, 3)], Naoto Suzuki[1), 3), 4)] Ryo Koyama-Nasu[1)], Daisuke Ogiya[5)], Kazuki Okuyama[1)], Yuichiro Yamamoto[1)], Masako Takamatsu[1)], Natsumi Kurosaki[1)], Kiyoshi Ando[5)], Akihiko Murata[6)], Masato Ohtsuka[7)], So Nakagawa[8, 9)], Koko Katagiri[3)], and Ai Kotani[1, 10, 11)]

[1]Department of Hematological Malignancy, Institute of Medical Science , Tokai University, Isehara, Kanagawa, Japan.

[2]Division of Clinical Oncology and Hematology, The Jikei University School of Medicine, Minato-ku, Tokyo, Japan.

[3]Department of Biosciences, School of Science, Kitasato University, Sagamihara, Kanagawa, Japan.

[4]Department of Computational Biology and Medical Sciences, Graduate School of Frontier Sciences, The University of Tokyo, Kashiwa, Chiba, Japan.

[5]Department of Hematology and Oncology, Tokai University School of Medicine, Isehara, Kanagawa, Japan.

[6]Division of Immunology, Department of Molecular and Cellular Biology, School of Life Science, Faculty of Medicine, Tottori University, Yonago, Tottori, Japan.

[7]Department of Molecular Life Science, Division of Basic Medical Science and Molecular Medicine, Tokai University School of Medicine, Isehara, Kanagawa, Japan

[8]Biomedical Informatics Laboratory, Department of Molecular Life Science, Tokai University School of Medicine, Isehara, Kanagawa, Japan.

[9]Micro/Nano Technology Center, Tokai University, Hiratsuka, Kanagawa, Japan.

[10]Precursory Research for Embryonic Science and Technology, Japan Science and Technology Agency, Saitama, Japan.

[11]AMED-PRIME, Japan Agency for Medical Research and Development, Tokyo, Japan.

Supplementary Figures S1 to S5 and Tables S1 to S8.

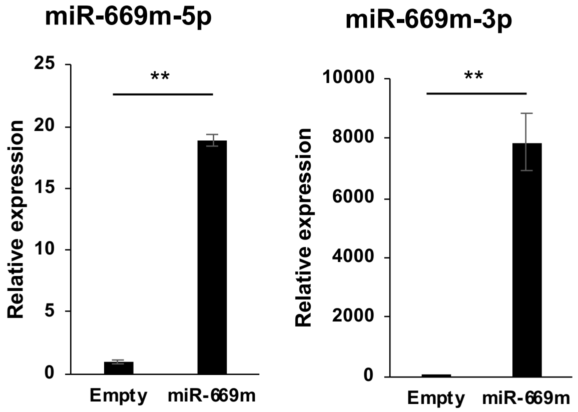

Fig. S1. miR-669m-transduced vector increased mature miRNAs, miR-669m-5p and miR-669m-3p.
Two mature miRNAs of miR-669m, miR-669m-5p and miR-669m-3p, were evaluated. HEK293T cells were transfected with pMSCV/Empty or pMSCV/miR-669m, and expressions were analyzed by qPCR. The error bars indicate SD (n = 3). ***p* < 0.01.

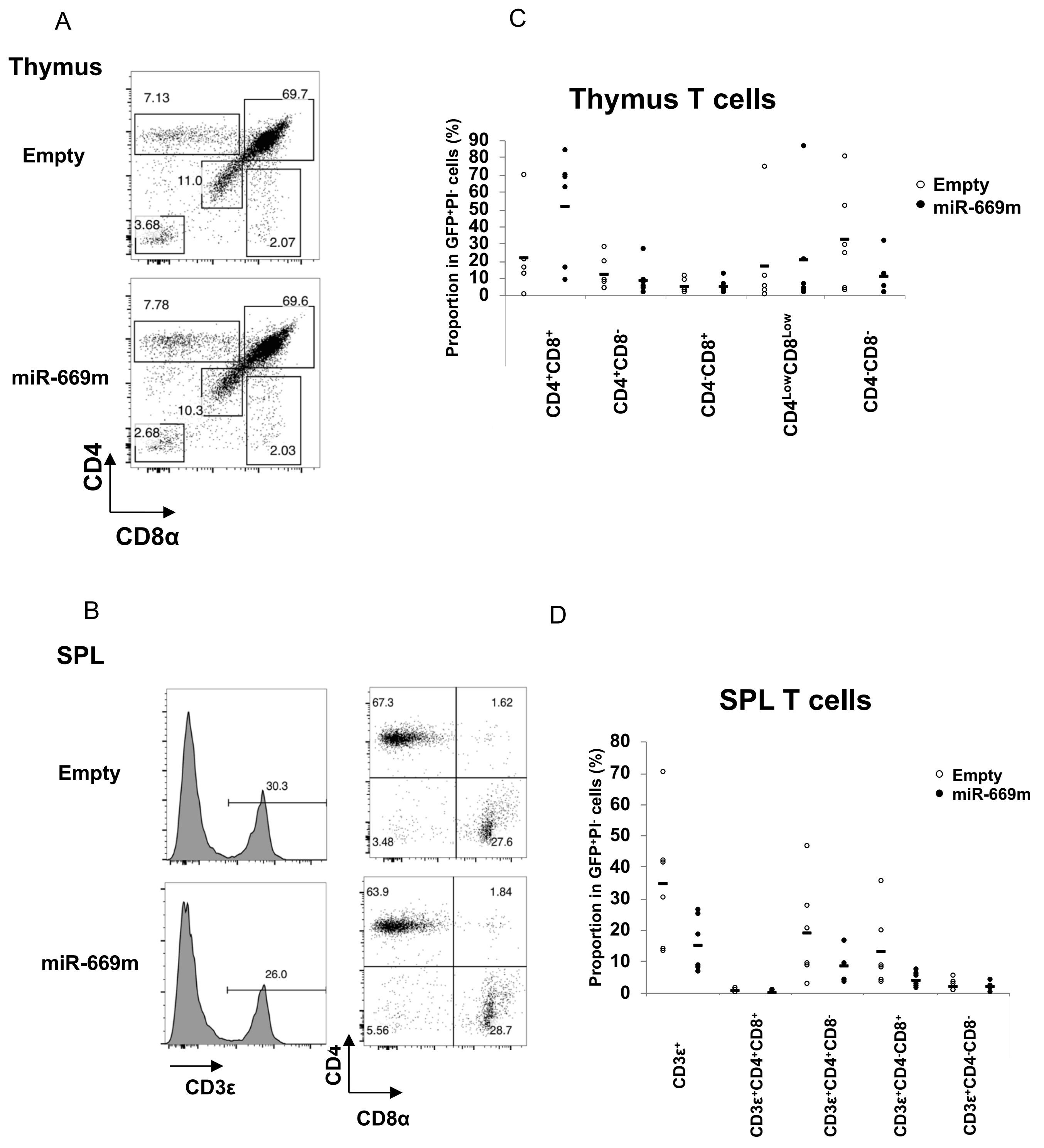

Fig. S2. miR-669m did not inhibit T cell differentiation *in vivo*.
Procedures were shown in Fig. 1A. (A)(B) Gating strategies for flow cytometry analysis. (A) Thymus of $GFP^{+}PI^{-}$ cells were analyzed by CD4/CD8α. (B) Spleen (SPL) of $CD3ε^{+}GFP^{+}PI^{-}$ cells were gated and then CD4/CD8α analysis was performed. (C)(D) Each fraction of control vector- or miR-669m- transduced cells are shown. Each dot represents data from one recipient mouse (n = 6).

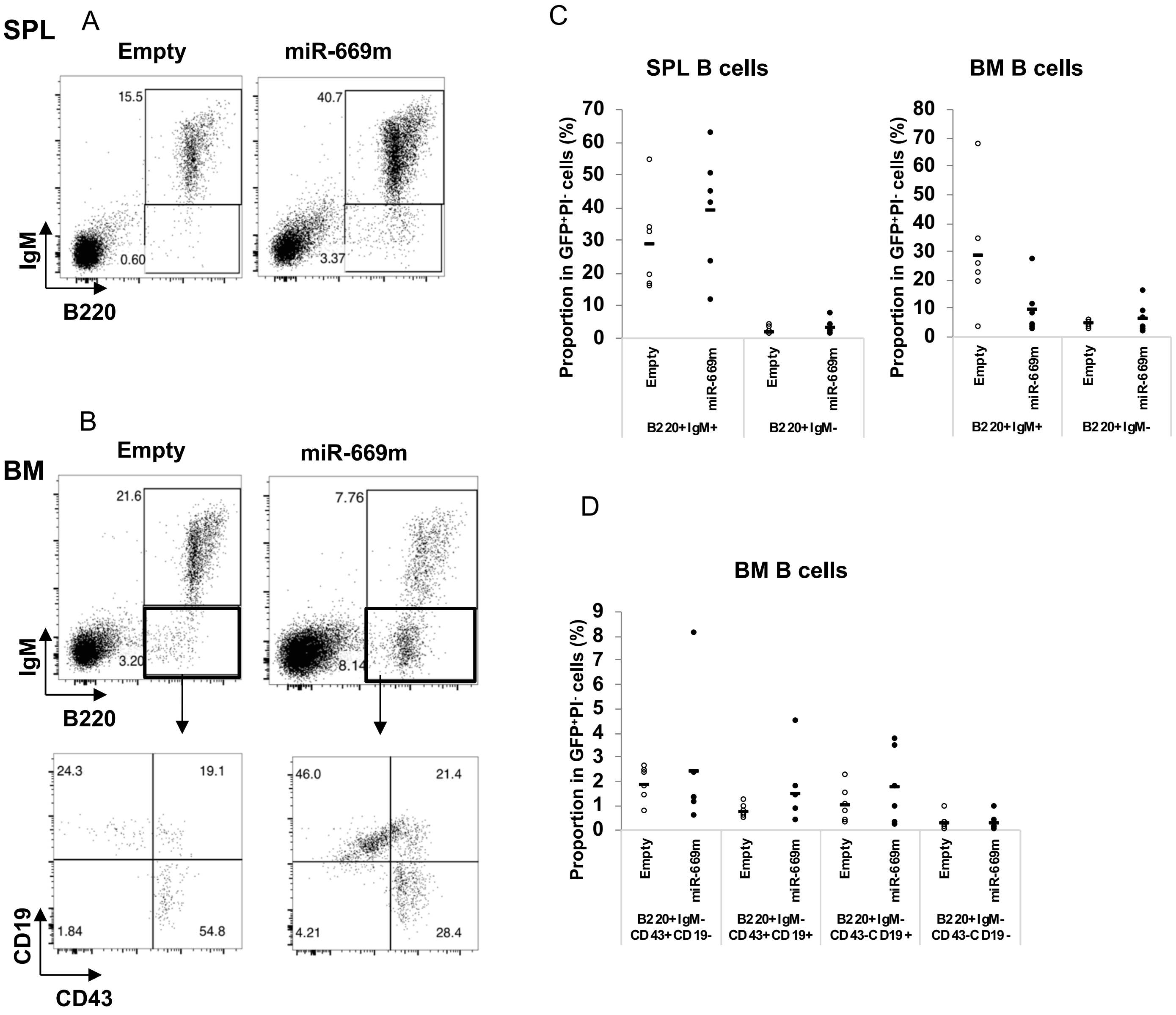

Fig. S3. miR-669m did not inhibit B cell differentiation *in vivo*.
Procedures were shown in Fig. 1A. (A)(B) Gating strategies for flow cytometry analysis. (A) Spleen (SPL) of GFP^+PI^- cells were analyzed by IgM/B220. (B) Bone Marrow (BM) of $B220^+IgM^+$ or $B220^+IgM^-$ GFP^+PI^- cells were gated and then CD19/CD43 analysis was performed. (C)(D) Each fraction of control vector- or miR-669m- transduced cells are shown. Each dot represents data from one recipient mouse (n = 6).

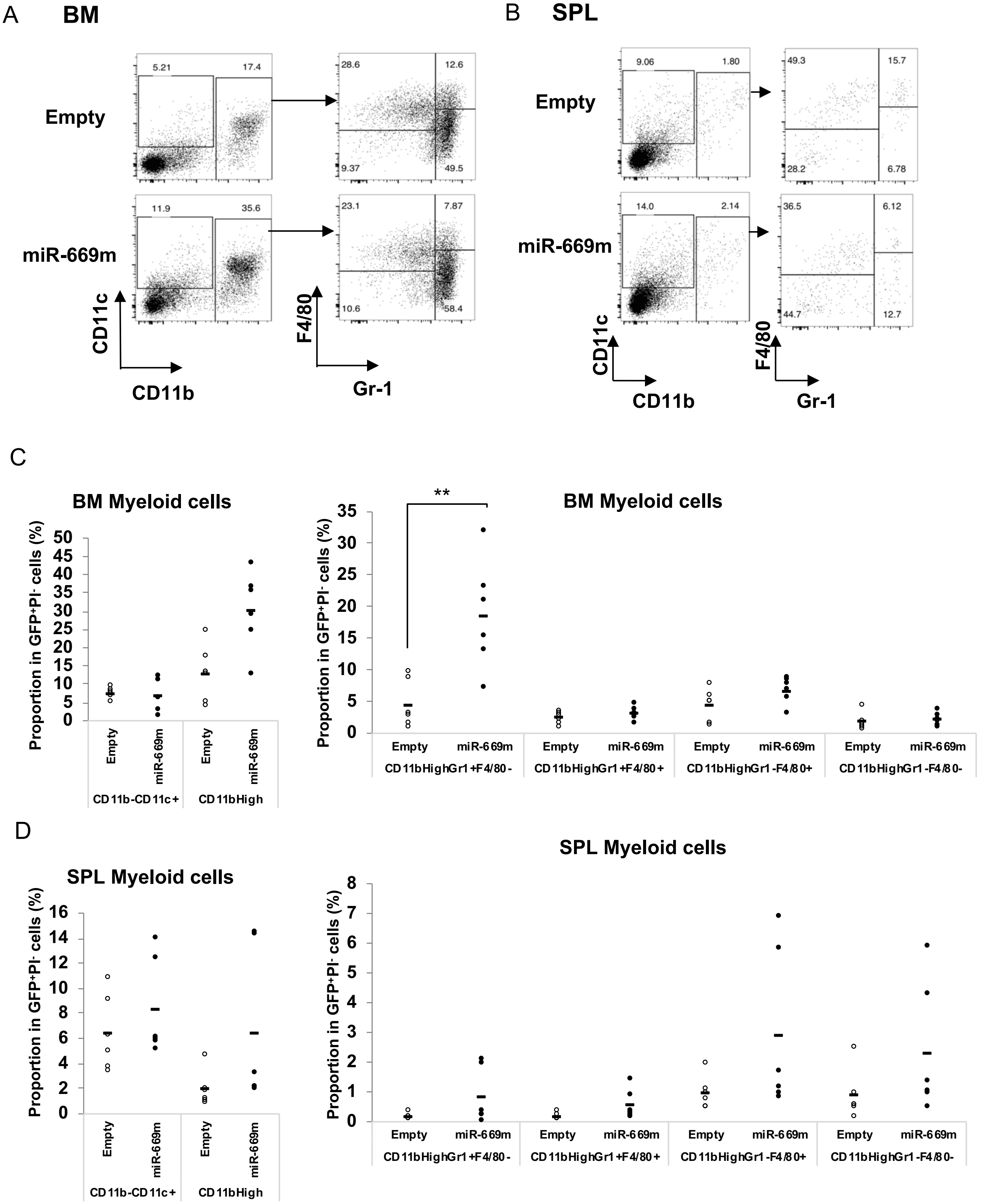

Fig. S4. miR-669m inhibits myeloid cell differentiation in bone marrow *in vivo*.
Procedures were shown in Fig. 1A. (A)(B) Gating strategies for flow cytometry analysis. Bone Marrow (BM) or Spleen (SPL) of CD11b-CD11c+ or CD11b^{high} GFP^{+}PI^{-} cells were gated. CD11b^{high} cells were further analyzed by Gr-1/F4/80. (C)(D) Each fraction of control vector- or miR-669m- transduced cells are shown. Each dot represents data from one recipient mouse (n = 6). ***p* < 0.01.

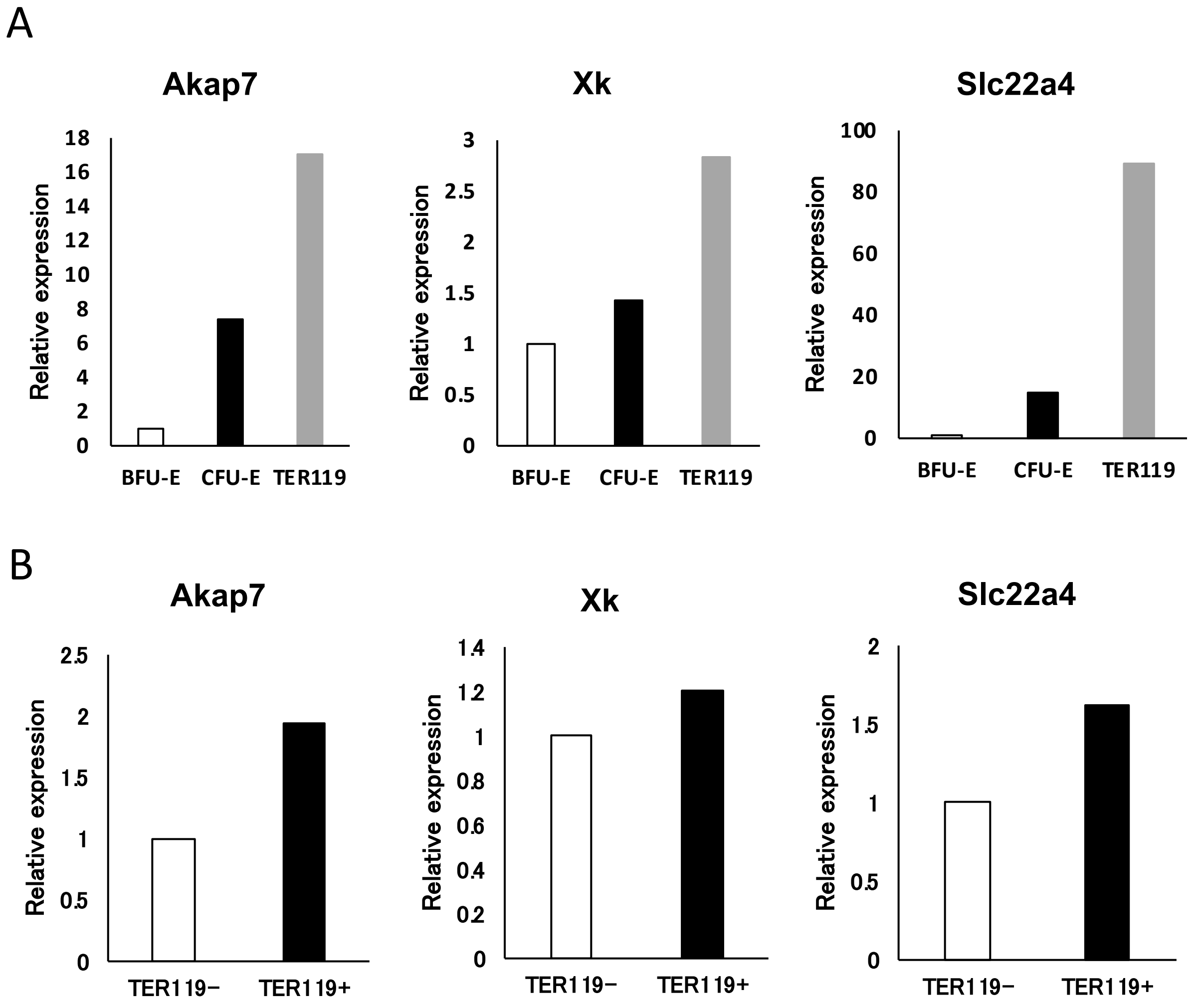

Fig. S5. RNA-seq data show that the target genes are upregulated progression of erythroid differentiation. FL erythroblast cells of previous RNA-seq data, (A) GSM640429-31 and (B) GSM1859519-20, were reanalyzed. Relative expression of Akap7, Xk, and Slc22a4 are shown. (A) BFU-E, CFU-E, and TER119^{+} cells, (B)TER119^{-} and TER119^{+} cells were evaluated.

Table S1. Candidate 24 targets of miR-669m suppressing erythroblast differentiation by Targetscan_v7.1.

GeneID	R2.RPKM	R3.RPKM	R4.RPKM	R5.RPKM
Cd59a	2.14	15.59	18.96	12.52
Igfbp1	0.41	2.01	0.53	3.08
Ccrl2	1.82	8.01	8.68	8.43
Aqp8	0.21	1.14	0.55	0.87
Gpr37	0	0.02	0.07	0.14
Slc22a4	11.18	35.87	33.26	23.65
Akap7	1.61	4.43	5.2	2.95
Xk	11.01	21.68	15.22	7.59
Gp9	7.83	19.67	8.53	11.54
Irx2	0.02	0.03	0.07	0.13
AI182371	0.1	0.14	0.14	0.04
Samd5	0	0.03	0.03	0.08
Cttnbp2	0.13	0.37	0.29	0.25
Inhbb	0.04	0.05	0.1	0.02
Elavl4	0	0.02	0.01	0.05
Rad51c	1.31	2.57	1.33	1.55
Gas1	0.07	0.16	0.11	0.2
Slc2a13	0	0.01	0.02	0.05
Havcr2	0.23	0.4	0.46	0.4
Car10	0	0.03	0.05	0.02
Lcor	0.33	0.42	0.38	0.22
Mobp	0	0.02	0.02	0.02
Mmp13	0	0.02	0.02	0.02
Avpr1a	0.02	0.03	0.03	0.02

Table S2. Known erythroid regulator genes were not target of miR-669m.

GeneID	Score (5p+3p)
Gata1	N/A
Gata2	N/A
Scl	N/A
Tal1	-0.01
Fog	N/A
Lmo2	N/A
Ldb1	N/A
Klf1	N/A
Riok3	0
Mxi1	0
Gcn5	N/A
Myb	0
Hdac2	N/A

N/A: not applicable

Table S3. Expression of miR-466-669 cluster in physiological erythroblasts (same dataset as Fig. 2)

	miRNA Name	R2.RPKM	R3.RPKM	R4.RPKM	R5.RPKM	Total
**Top 5 miRNAs**	mmu-miR-466i-5p	9944	10665	9768	11937	42314
	mmu-miR-3470b	1999	1461	1120	1234	5814
	mmu-miR-3470a	1489	1073	804	929	4295
	mmu-miR-692	494	799	1029	1579	3901
	mmu-miR-3473b	514	461	274	307	1556
	//	//	//	//	//	//
**miR-466-669 cluster (miR-669m)**	mmu-miR-466f-3p	80	71	41	75	267
	mmu-miR-466m-5p	13	9	14	13	49
	**mmu-miR-669m-5p**	13	9	14	13	49
	mmu-miR-669f-5p	13	12	12	11	48
	mmu-miR-669p-3p	2	3	6	4	15
	mmu-miR-466f	3	4	1	4	12
	mmu-miR-669c-3p	5	1	0	4	10
	mmu-miR-466m-3p	1	4	0	3	8
	mmu-miR-669h-3p	0	0	1	3	4
	mmu-miR-466c-5p	0	0	0	3	3
	mmu-miR-466g	0	0	0	3	3
	mmu-miR-669i	2	0	0	1	3
	mmu-miR-297c-5p	0	0	0	2	2
	mmu-miR-466h-5p	1	1	0	0	2
	mmu-miR-297a-5p	0	0	1	0	1
	mmu-miR-669b-3p	0	1	0	0	1
	mmu-miR-669b-5p	0	0	0	1	1
	mmu-miR-669d-5p	0	1	0	0	1
	mmu-miR-669k-5p	0	0	1	0	1
	mmu-miR-297a-3p	0	0	0	0	0
	mmu-miR-297b-3p	0	0	0	0	0
	mmu-miR-297b-5p	0	0	0	0	0
	mmu-miR-297c-3p	0	0	0	0	0
	mmu-miR-466a-3p	0	0	0	0	0
	mmu-miR-466a-5p	0	0	0	0	0
	mmu-miR-466b-3p	0	0	0	0	0
	mmu-miR-466b-5p	0	0	0	0	0
	mmu-miR-466c-3p	0	0	0	0	0
	mmu-miR-466d-3p	0	0	0	0	0
	mmu-miR-466d-5p	0	0	0	0	0
	mmu-miR-466e-3p	0	0	0	0	0
	mmu-miR-466e-5p	0	0	0	0	0
	mmu-miR-466f-5p	0	0	0	0	0
	mmu-miR-466h-3p	0	0	0	0	0
	mmu-miR-466l-3p	0	0	0	0	0
	mmu-miR-466l-5p	0	0	0	0	0
	mmu-miR-466n-3p	0	0	0	0	0
	mmu-miR-466n-5p	0	0	0	0	0
	mmu-miR-466o-3p	0	0	0	0	0
	mmu-miR-466o-5p	0	0	0	0	0
	mmu-miR-466p-3p	0	0	0	0	0
	mmu-miR-466p-5p	0	0	0	0	0
	mmu-miR-466q	0	0	0	0	0
	mmu-miR-467a-3p	0	0	0	0	0
	mmu-miR-467a-5p	0	0	0	0	0
	mmu-miR-467b-3p	0	0	0	0	0
	mmu-miR-467b-5p	0	0	0	0	0
	mmu-miR-467c-3p	0	0	0	0	0
	mmu-miR-467c-5p	0	0	0	0	0
	mmu-miR-467d-3p	0	0	0	0	0
	mmu-miR-467d-5p	0	0	0	0	0
	mmu-miR-467e-3p	0	0	0	0	0
	mmu-miR-467e-5p	0	0	0	0	0
	mmu-miR-669a-3-3p	0	0	0	0	0
	mmu-miR-669a-3p	0	0	0	0	0
	mmu-miR-669a-5p	0	0	0	0	0
	mmu-miR-669c-5p	0	0	0	0	0
	mmu-miR-669d-2-3p	0	0	0	0	0
	mmu-miR-669d-3p	0	0	0	0	0
	mmu-miR-669e-3p	0	0	0	0	0
	mmu-miR-669e-5p	0	0	0	0	0
	mmu-miR-669f-3p	0	0	0	0	0
	mmu-miR-669g	0	0	0	0	0
	mmu-miR-669h-5p	0	0	0	0	0
	mmu-miR-669j	0	0	0	0	0
	mmu-miR-669k-3p	0	0	0	0	0
	mmu-miR-669l-3p	0	0	0	0	0
	mmu-miR-669l-5p	0	0	0	0	0
	**mmu-miR-669m-3p**	0	0	0	0	0
	mmu-miR-669o-3p	0	0	0	0	0
	mmu-miR-669o-5p	0	0	0	0	0
	mmu-miR-669p-5p	0	0	0	0	0

Table S4. Expression of miR-466-669 cluster in physiological erythroblasts (same dataset as Fig. S5A)

	miRNA Name	BFU-E	CFU-E	TER119	Total
**Top 5 miRNAs**	mmu-miR-466i-5p	3060	2431.5	5596.5	11088
	mmu-miR-3470b	700.5	410.5	977.5	2088.5
	mmu-miR-703	811	752.5	188	1751.5
	mmu-miR-3470a	551.5	268	733	1552.5
	mmu-miR-692	189	334	824	1347
	//	//	//	//	//
**miR-466-669 cluster (miR-669m)**	mmu-miR-466f-3p	28	22	63	113
	mmu-miR-466m-5p	3	2.5	5.5	11
	**mmu-miR-669m-5p**	3	2.5	5.5	11
	mmu-miR-669f-5p	3.5	2.5	3	9
	mmu-miR-669c-3p	1.5	1.5	3	6
	mmu-miR-466f	1	1.5	3	5.5
	mmu-miR-466m-3p	0	1	1.5	2.5
	mmu-miR-466a-5p	1	1	0	2
	mmu-miR-466e-5p	1	1	0	2
	mmu-miR-669d-5p	0	0	2	2
	mmu-miR-669i	1.5	0	0	1.5
	mmu-miR-466g	0.5	0.5	0	1
	mmu-miR-669o-5p	0	1	0	1
	mmu-miR-297a-5p	0	0.5	0	0.5
	mmu-miR-297c-5p	0.5	0	0	0.5
	mmu-miR-466f-5p	0	0.5	0	0.5
	mmu-miR-669f-3p	0	0.5	0	0.5
	mmu-miR-669h-3p	0.5	0	0	0.5
	mmu-miR-669l-5p	0.5	0	0	0.5
	mmu-miR-669p-3p	0	0	0.5	0.5
	mmu-miR-297a-3p	0	0	0	0
	mmu-miR-297b-3p	0	0	0	0
	mmu-miR-297b-5p	0	0	0	0
	mmu-miR-297c-3p	0	0	0	0
	mmu-miR-466a-3p	0	0	0	0
	mmu-miR-466b-3p	0	0	0	0
	mmu-miR-466b-5p	0	0	0	0
	mmu-miR-466c-3p	0	0	0	0
	mmu-miR-466c-5p	0	0	0	0
	mmu-miR-466d-3p	0	0	0	0
	mmu-miR-466d-5p	0	0	0	0
	mmu-miR-466e-3p	0	0	0	0
	mmu-miR-466h-3p	0	0	0	0
	mmu-miR-466h-5p	0	0	0	0
	mmu-miR-466l-3p	0	0	0	0
	mmu-miR-466l-5p	0	0	0	0
	mmu-miR-466n-3p	0	0	0	0
	mmu-miR-466n-5p	0	0	0	0
	mmu-miR-466o-3p	0	0	0	0
	mmu-miR-466o-5p	0	0	0	0
	mmu-miR-466p-3p	0	0	0	0
	mmu-miR-466p-5p	0	0	0	0
	mmu-miR-466q	0	0	0	0
	mmu-miR-467a-3p	0	0	0	0
	mmu-miR-467a-5p	0	0	0	0
	mmu-miR-467b-3p	0	0	0	0
	mmu-miR-467b-5p	0	0	0	0
	mmu-miR-467c-3p	0	0	0	0
	mmu-miR-467c-5p	0	0	0	0
	mmu-miR-467d-3p	0	0	0	0
	mmu-miR-467d-5p	0	0	0	0
	mmu-miR-467e-3p	0	0	0	0
	mmu-miR-467e-5p	0	0	0	0
	mmu-miR-669a-3-3p	0	0	0	0
	mmu-miR-669a-3p	0	0	0	0
	mmu-miR-669a-5p	0	0	0	0
	mmu-miR-669b-3p	0	0	0	0
	mmu-miR-669b-5p	0	0	0	0
	mmu-miR-669c-5p	0	0	0	0
	mmu-miR-669d-2-3p	0	0	0	0
	mmu-miR-669d-3p	0	0	0	0
	mmu-miR-669e-3p	0	0	0	0
	mmu-miR-669e-5p	0	0	0	0
	mmu-miR-669g	0	0	0	0
	mmu-miR-669h-5p	0	0	0	0
	mmu-miR-669j	0	0	0	0
	mmu-miR-669k-3p	0	0	0	0
	mmu-miR-669k-5p	0	0	0	0
	mmu-miR-669l-3p	0	0	0	0
	**mmu-miR-669m-3p**	0	0	0	0
	mmu-miR-669o-3p	0	0	0	0
	mmu-miR-669p-5p	0	0	0	0

Table S5. Expression of miR-466-669 cluster in physiological erythroblasts (same dataset as Fig. S5B)

	miRNA Name	TER119-	TER119+	total
Top 5 miRNAs	mmu-miR-466i-5p	9849	8870	18719
	mmu-miR-703	13862	3882	17744
	mmu-miR-6240	5177	8155	13332
	mmu-miR-692	4163	6859	11022
	mmu-miR-3470b	5781	4120	9901
	//	//	//	//
miR-466-669 cluster (miR-669m)	mmu-miR-466f-3p	46	98	144
	mmu-miR-466m-5p	8	9	17
	**mmu-miR-669m-5p**	8	9	17
	mmu-miR-669f-5p	7	7	14
	mmu-miR-297c-5p	3	8	11
	mmu-miR-466f	4	6	10
	mmu-miR-669c-3p	2	5	7
	mmu-miR-467a-3p	5	1	6
	mmu-miR-467d-3p	5	1	6
	mmu-miR-297a-5p	1	3	4
	mmu-miR-466a-5p	2	2	4
	mmu-miR-669d-5p	0	4	4
	mmu-miR-466p-5p	2	1	3
	mmu-miR-466f-5p	0	2	2
	mmu-miR-669b-3p	1	1	2
	mmu-miR-466e-5p	0	1	1
	mmu-miR-466g	1	0	1
	mmu-miR-466h-5p	0	1	1
	mmu-miR-466m-3p	0	1	1
	mmu-miR-669f-3p	1	0	1
	mmu-miR-669h-3p	1	0	1
	mmu-miR-669i	0	1	1
	mmu-miR-669p-3p	0	1	1
	mmu-miR-297a-3p	0	0	0
	mmu-miR-297b-3p	0	0	0
	mmu-miR-297b-5p	0	0	0
	mmu-miR-297c-3p	0	0	0
	mmu-miR-466a-3p	0	0	0
	mmu-miR-466b-3p	0	0	0
	mmu-miR-466b-5p	0	0	0
	mmu-miR-466c-3p	0	0	0
	mmu-miR-466c-5p	0	0	0
	mmu-miR-466d-3p	0	0	0
	mmu-miR-466d-5p	0	0	0
	mmu-miR-466e-3p	0	0	0
	mmu-miR-466h-3p	0	0	0
	mmu-miR-466l-3p	0	0	0
	mmu-miR-466l-5p	0	0	0
	mmu-miR-466n-3p	0	0	0
	mmu-miR-466n-5p	0	0	0
	mmu-miR-466o-3p	0	0	0
	mmu-miR-466o-5p	0	0	0
	mmu-miR-466p-3p	0	0	0
	mmu-miR-466q	0	0	0
	mmu-miR-467a-5p	0	0	0
	mmu-miR-467b-3p	0	0	0
	mmu-miR-467b-5p	0	0	0
	mmu-miR-467c-3p	0	0	0
	mmu-miR-467c-5p	0	0	0
	mmu-miR-467d-5p	0	0	0
	mmu-miR-467e-3p	0	0	0
	mmu-miR-467e-5p	0	0	0
	mmu-miR-669a-3-3p	0	0	0
	mmu-miR-669a-3p	0	0	0
	mmu-miR-669a-5p	0	0	0
	mmu-miR-669b-5p	0	0	0
	mmu-miR-669c-5p	0	0	0
	mmu-miR-669d-2-3p	0	0	0
	mmu-miR-669d-3p	0	0	0
	mmu-miR-669e-3p	0	0	0
	mmu-miR-669e-5p	0	0	0
	mmu-miR-669g	0	0	0
	mmu-miR-669h-5p	0	0	0
	mmu-miR-669j	0	0	0
	mmu-miR-669k-3p	0	0	0
	mmu-miR-669k-5p	0	0	0
	mmu-miR-669l-3p	0	0	0
	mmu-miR-669l-5p	0	0	0
	**mmu-miR-669m-3p**	0	0	0
	mmu-miR-669o-3p	0	0	0
	mmu-miR-669o-5p	0	0	0
	mmu-miR-669p-5p	0	0	0

Table S6. Expression of Sfmbt2 gene in physiological erythroblasts (same dataset as Fig. 2)

	R2	R3	R4	R5
Sfmbt2	0.216478	0.008956	0.021424	0.075927

Table S7. Expression of Sfmbt2 gene in physiological erythroblasts (same dataset as Fig. S5A)

	BFU-E	CFU-E	TER119
Sfmbt2	0.620521	0.087698	0

Table S8. Expression of Sfmbt2 gene in physiological erythroblasts (same dataset as Fig. S5B)

	TER119-	TER119+
Sfmbt2	0.080303	0.032229
